# The relationship between size and longevity of the malaria vector *Anopheles gambiae* (*s.s.*) depends on the larval environment

**DOI:** 10.1186/s13071-018-3058-3

**Published:** 2018-08-29

**Authors:** Antoine M. G. Barreaux, Chris M. Stone, Priscille Barreaux, Jacob C. Koella

**Affiliations:** 1 Center for Infectious Disease Dynamics and Department of Entomology, Pennsylvania State University, University Park, PA 16802 USA; 2 Laboratory of Ecology and Epidemiology of Parasites, Institute of Biology, University of Neuchâtel, Rue Emile-Argand 11, 2000 Neuchâtel, Switzerland; 3 Illinois Natural History Survey, University of Illinois, Champaign, IL 61820 USA

**Keywords:** *Anopheles gambiae*, Body size, Larval environment, Longevity, Mosquito life history, Piece-wise structural equation modelling

## Abstract

**Background:**

Understanding the variation in vector-borne disease transmission intensity across time and space relies on a thorough understanding of the impact of environmental factors on vectorial capacity traits of mosquito populations. This is driven primarily by variation in larval development and growth, with carryover effects influencing adult traits such as longevity and adult body size. The relationship between body size and longevity strongly affects the evolution of life histories and the epidemiology of vector-borne diseases. This relationship ranges from positive to negative but the reasons for this variability are not clear. Both traits depend on a number of environmental factors, but primarily on temperature as well as availability of nutritional resources. We therefore asked how the larval environment of the mosquito *Anopheles gambiae* Giles (*sensu stricto*) (Diptera: Culicidae) affects the relationship between body size and longevity.

**Methods:**

We reared the larvae of *An. gambiae* individually at three temperatures (21, 25 and 29 °C) and two food levels (the standard and 50% of our laboratory diet) and measured adult size and longevity. We estimated the direct and indirect (*via* adult size) effects of food and temperature on longevity with a piecewise structural equation model (SEM).

**Results:**

We confirmed the direct effects of food and temperature during larval development on body size, as wing length decreased with increasing temperature and decreasing food levels. While the overall relationship between size and longevity was weak, we measured striking differences among environments. At 25 °C there was no clear relationship between size and longevity; at 29 °C the association was negative with standard food but positive with low food; whereas at 21 °C it was positive with standard food but negative with low food.

**Conclusions:**

The larval environment influences the adult’s fitness in complex ways with larger mosquitoes living longer in some environments but not in others. This confirmed our hypothesis that the relationship between size and longevity is not limited to a positive correlation. A better understanding of this relationship and its mechanisms may improve the modelling of the transmission of vector borne diseases, the evolution of life history traits, and the influence of vector control.

**Electronic supplementary material:**

The online version of this article (10.1186/s13071-018-3058-3) contains supplementary material, which is available to authorized users.

## Background

Understanding the variation in vector-borne disease transmission intensity across time and space relies on a thorough understanding of the effect of environmental factors on vectorial capacity traits of mosquito populations. A major area of interest, for instance, has been on understanding the effect of temperature on vectorial capacity [1], although a wide range of environmental and anthropogenic factors impacts mosquito populations [2].

Fluctuations in mosquito population size are driven primarily by variation in larval development and growth, with direct consequences for vector-borne disease transmission. Additionally, carryover effects from the larval stage can influence vectorial capacity traits such as longevity, fertility, vector competence and biting behavior [3–5].

Larval growth and development of mosquitoes, as well as life history traits such as the body size of adults, depends on a number of environmental factors, but primarily on temperature as well as availability of nutritional resources (whether due to lower abundance in the habitat or resulting from resource competition) [4, 6–9]. Considering just the impacts on larval growth, foraging behavior, and survival of several of these factors simultaneously can quickly become complex [7, 10–13]. The consequences of such interactions between extrinsic factors experienced during the larval stage on adult traits has received much less attention but becomes important if one wishes to move beyond considering the effect of one environmental variable in isolation, to predictions under field conditions.

For epidemiological models to consider more than one environmental variable at the time, it would be useful if the combined effects of temperature and larval resource quality or density could be considered through a shared metric. Similar approaches have been used to great effect in a variety of systems where a continuous trait, such as body size, has a strong relation to a number of life history traits and individual fitness [14]. In mosquitoes, adult body size is affected by temperature and resource levels and could putatively play such a role. We know relatively little however of the relation between mosquito body size and adult longevity, particularly whether variation in size resulting from different extrinsic factors leads to different outcomes. We focus on longevity as it is a major determinant of evolutionary fitness [15]. In mosquitoes, longevity, along with the biting rate, has the strongest influence on vectorial capacity [16, 17].

We expect longevity to be positively correlated with body size as a larger mosquito should emerge with larger teneral reserves [18] and larger mosquitoes are also more efficient in accumulating reserves from blood meals [19]. While it is true that longevity often increases with body size [19, 20], this correlation is not always apparent [4, 21] and can even be negative [9, 22]. One of the reasons for this variation may be that survival and adult body size respond differently to environmental factors. For example, undernourished juveniles generally become small adults [23] with increased longevity [9, 24], so that we expect a negative correlation between the two traits among environments. In contrast, colder temperature generally leads to larger adults [7] that live longer, in cold-blooded animals [25], giving a positive correlation. We expect the slope of the correlation between adult body size and longevity to change depending on the larval environment and we do not know how the interaction of two different environmental factors, larval diet and larval temperature, may influence this correlation of two traits that respond differently to these environmental factors.

We more formally investigate how two environmental factors (temperature and nutrition levels during larval development) that both influence adult size and longevity, affect the association between these two traits of epidemiological relevance in an important vector of human malaria, *Anopheles gambiae*. To do so, we reared *An. gambiae* mosquitoes at three different larval temperatures (21, 25 and 29 °C) and two food levels (the standard and 50% of our laboratory diet), let the adult females blood feed and measured their size and their longevity.

## Methods

Newly hatched larvae from our Kisumu colony of *Anopheles gambiae* Giles (*s.s.*), were placed individually in 12-well-plates (VWR International S.a.r.l., Nyon, Switzerland), with each well containing one larva in 3 ml of deionised water. The well-plates were placed inside incubators and the larvae were reared at constant temperatures, at 21, 25 or 29 °C, 70 ± 5% RH and a 12:12 h light:dark cycle. The larvae were fed our standard diet or 50% of it. Each larva received daily 100 μl of a solution of water and fish food with the amount of food varying over the days. The standard diet was on hatching day: 0.04 mg Tetramin^TM^ baby fish food (Qualipet, Neuchatel, Switzerland) per larva; 1 day after hatching: 0.06 mg; 2 days: 0.08 mg; 3 days: 0.16 mg; 4 days: 0.32 mg, 5 days or later: 0.6 mg [26].

Each pupa was put into a 180 ml plastic cup (VWR International S.a.r.l., Nyon, Switzerland) covered with netting. Emerged females had access to 10% sugar solution and were held in an insectary at 26 ± 1 °C, 70 ± 5% RH and a 12:12 h light:dark cycle, before the blood meal.

### Blood meal

Four to five days after emergence, females were given the opportunity to feed on a mouse for 10 min. The mice were obtained from the Institute of Cell Biology, University of Bern, Switzerland, and had been anaesthetized by intra-peritoneal injection of 8.5 ml/kg mix of Xylazine Xylasol® (20 mg/ml), Ketamine Ketasol® (100 mg/ml) and phosphate buffered saline (PBS). One day after the blood meal, individual fully engorged mosquitoes (187 out of 303) were transferred into cups with 10% sugar solution and then, for logistical reasons, moved to an insectary at 19 ± 1 °C.

### Longevity and body size

Longevity was measured as the time between the blood meal and death, which was assessed every 24 h. Survival after a first blood meal is not the same as lifespan from birth but taking a bloodmeal is a realistic behavior. Besides, one of the key factors in vectorial capacity is how long a mosquito can survive after a potential infectious blood meal, which has motivated a history of interest and studies on mosquitoes’ longevity post-blood meal. Larger individuals are more efficient at accumulating reserves used for fecundity, but blood meals are also used to synthesize lipids, which are used by female *An. gambiae* both for flight as well as for survival [19, 27].

Wing length was used as a proxy for body size. Both wings of each individual were measured from the tip to the distal end of the alula [28] with the software Image J (version 1.47f7); we used the mean length of the two wings in our analyses.

### Analyses

#### Effect of the larval environment on the time to pupation

We analysed the time to pupation of female mosquitoes depending on the larval environment by using a gaussian generalised linear model (GLM) of time to pupation regarding the larval diet and the larval temperature and the interaction between these factors.

#### Relationship between wing length and survival

We analysed how wing length was related to longevity depending on the larval environment. As body size is in part determined by the environment, and potentially associated with longevity, we ran our analysis in two steps.

We first analysed the direct effects of the environment on wing length with an ANOVA and on longevity with a Cox proportional hazards survival analysis, including temperature, food and their interaction as factors for both analyses.

We then did a separate analysis where we estimated the direct and indirect (*via* wing length) effects of food and temperature on longevity in a single model with a piece-wise structural equation model (SEM) [29]. This approach relies on a set of linear equations that are evaluated individually in the model and that are described below. This recent SEM method, and the associated R package, enable the use of the current statistical methods like generalised linear models or mixed effects models in the classical causal network of SEM. Worked examples and a detailed description of how this approach differs from classical SEM methods can be found in a recent paper [29]. It is different to classical SEM methods that are based on covariances among variables.

In our first analysis wing length was not affected by an interaction of food and temperature (see results), so we modelled the direct effects of the environment on wing length. We analysed wing length regarding the larval diet and the larval temperature without interactions between these factors. This is expressed as the following equation:

1 $$ wing\ length\sim food+ temperature $$

Since we were interested in how food and temperature influenced the relationship between body size and longevity, we analysed longevity regarding the interactions between larval diet, larval temperature and wing length. Food and temperature were considered as factors and wing length a covariate. It gives the following equation:

2 $$ longevity\sim wing\ length\ast food\ast temperature $$

Analyses were done in R version 3.4.1. The SEM was implemented with the *piecewiseSEM* package [29].

## Results

### Time to pupation

**Fig. 1:**
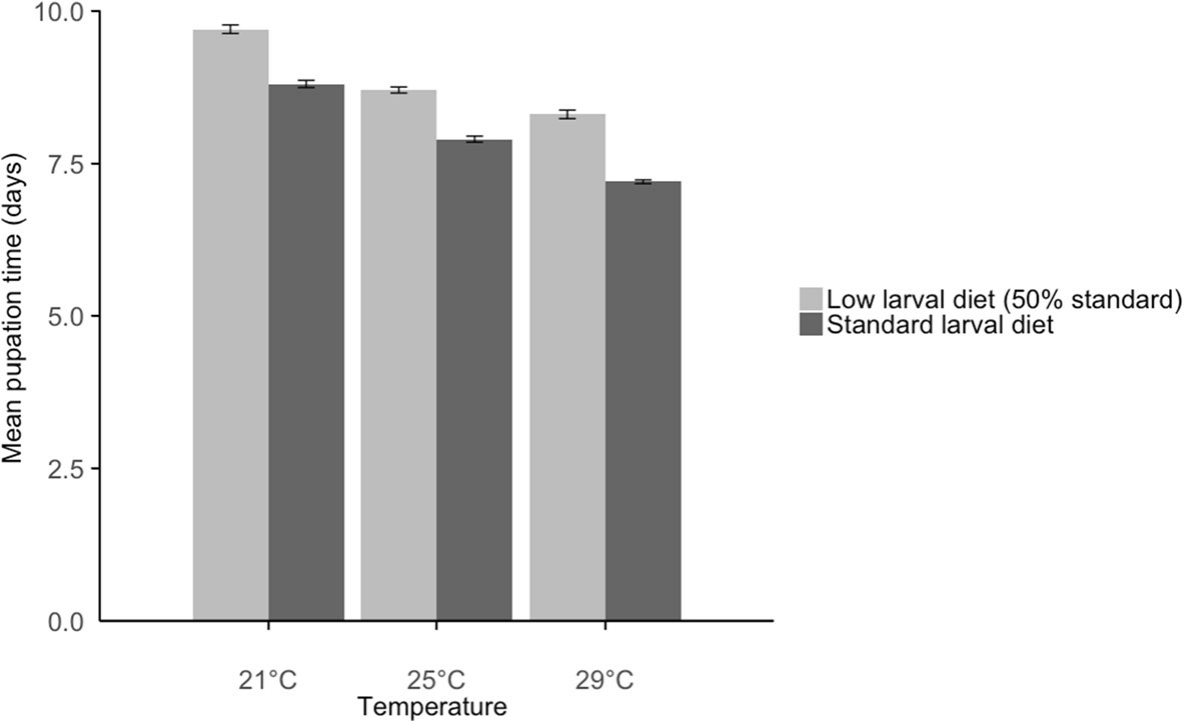
Mean female mosquito pupation time (in days) ± standard error, by larval rearing temperature (21, 25 and 29 °C) and level of larval nutrition (low, standard). The standard diet was on hatching day: 0.04 mg Tetramin baby fish food per larva; 1 day after hatching: 0.06 mg; 2 days: 0.08 mg; 3 days: 0.16 mg; 4 days: 0.32 mg; 5 days or later: 0.6 mg. The low diet was 50% of the standard. Larvae were reared at constant temperatures, at 21, 25 or 29 °C, 70 ± 5% RH and a 12:12 h light:dark cycle

The mean time to pupation decreased with the increase in temperature (*χ*^2^ = 612.09, *df* = 1, *P* < 0.0001), from 9.2 ± 0.05 (mean ± standard error, SE) days at 21 °C, to 8.3 ± 0.04 days at 25 °C and 7.8 ± 0.05 days at 29 °C (Fig. 1).

The mean time to pupation increased when the larval diet decreased by 50% (low diet) (*χ*^2^ = 392.30, *df* = 1, *P* < 0.0001), from 8.0 ± 0.04 days at standard diet to 8.85 ± 0.05 days at low diet (Fig. 1).

There was also a significant interaction between the effect of temperature and larval diet (Table 1 and Fig. 1), (*χ*^2^ = 6.61, *df* = 1, *P* = 0.01). The pupation time increased by nearly 2.5 days between 29 °C and standard food, and 21 °C and low food (respectively 7.17 ± 0.03 days and 9.65 ± 0.07 days). If only one environmental variable was modified (just food or just temperature) then the variation in pupation time was only up to 1.4 days. Modifying both the temperature and the food levels changed the pupation time more than modifying only one of the environmental factors.

**Table 1:** Mean pupation time (in days) per larval temperature and larval diet

Larval T (°C)	Larval diet	Mean pupation time (days)	SE
21	Standard	8.8	0.06
21	Low	9.7	0.07
25	Standard	7.9	0.05
25	Low	8.7	0.05
29	Standard	7.2	0.03
29	Low	8.3	0.07

*Abbreviations*: *SE* standard error, *T* temperature

### Relationship between longevity and size

**Fig. 2:**
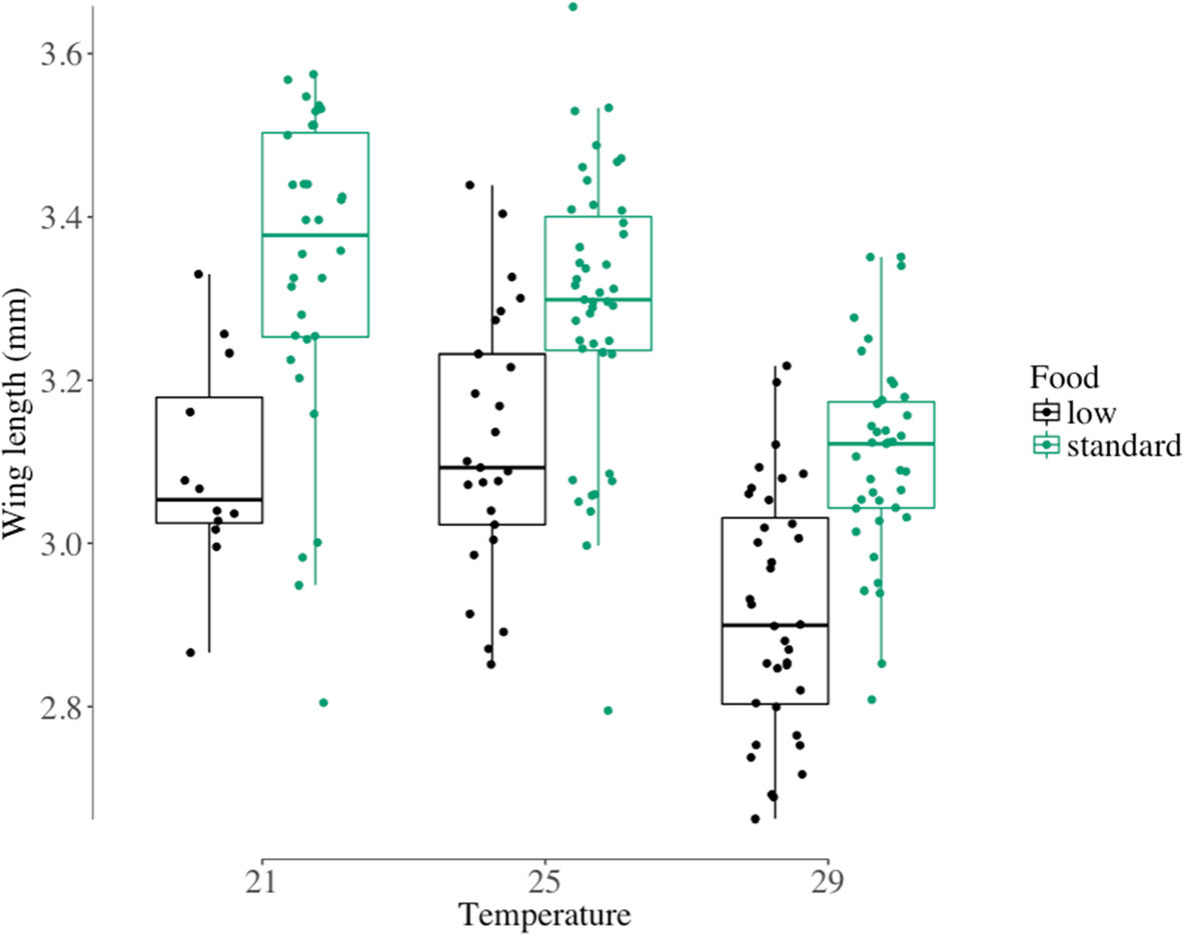
Distribution of female mosquito wing length (in mm) by larval rearing temperature (21, 25 and 29 °C) and level of larval nutrition (low, standard). The standard diet was on hatching day: 0.04 mg Tetramin baby fish food per larva; 1 day after hatching: 0.06 mg; 2 days: 0.08 mg; 3 days: 0.16 mg; 4 days: 0.32 mg; 5 days or later: 0.6 mg. The low diet was 50% of the standard. Larvae were reared at constant temperatures, at 21, 25 or 29 °C, 70 ± 5% RH and a 12:12 h light:dark cycle

Mosquitoes reared at half the standard larval diet had shorter wings (3.02 ± 0.021 mm) than those with the standard diet (3.24 ± 0.018 mm) (*F*_(1, 181)_ = 90.1, *P* < 0.0001). Wing length decreased with increasing temperature from 3.27 mm (± 0.032) at 21 °C, to 3.23 mm (± 0.022) at 25 °C, and 3.02 mm (± 0.019) at 29 °C (*F*_(2, 181)_ = 35.7, *P* < 0.0001). The effect of temperature was similar at the two food levels (Fig. 2) so the interaction between food and temperature was not significant (*F*_(2, 181)_ = 0.7, *P* = 0.49).

**Fig. 3:**
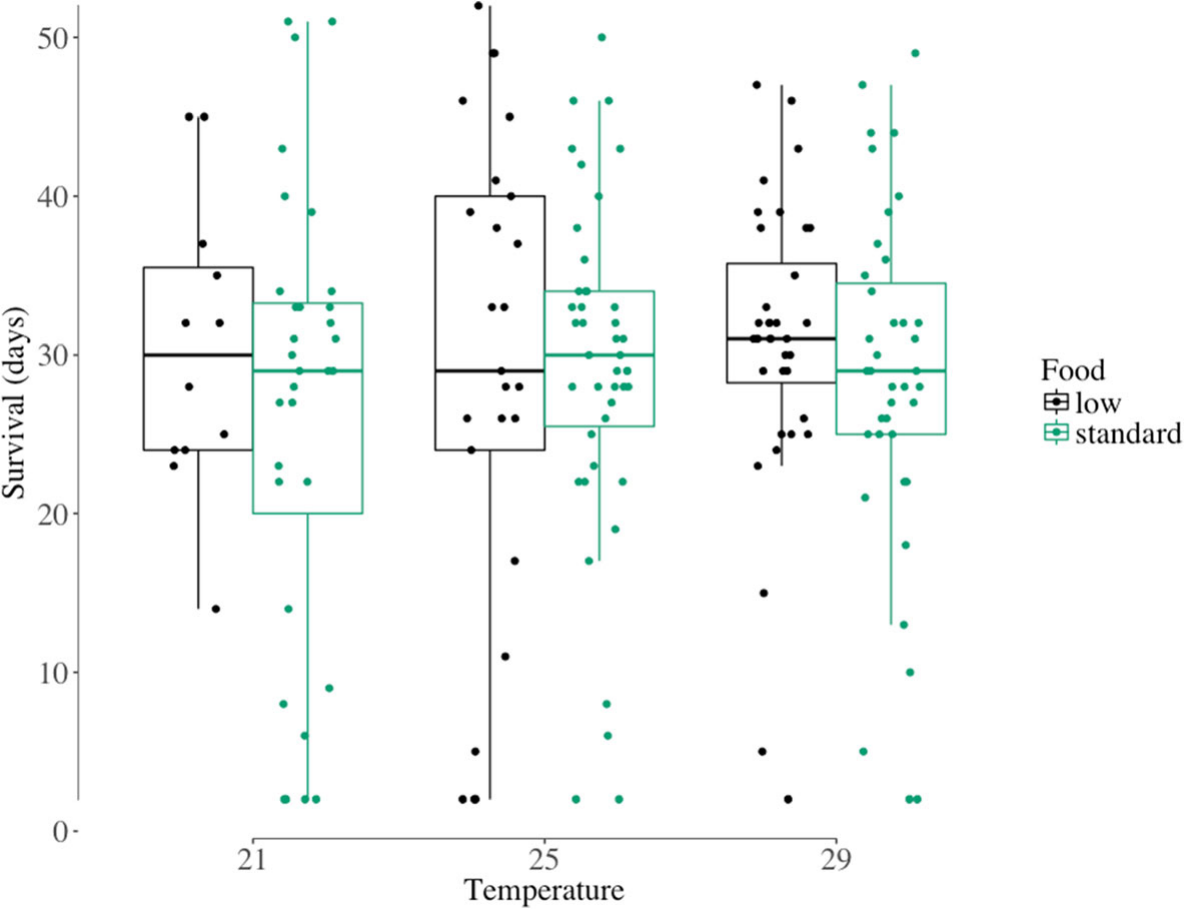
Distribution of female mosquito longevity (in days) by larval rearing temperature (21, 25 and 29 °C) and level of larval nutrition (low, standard). Longevity is here the number of days between the blood meal and death for each female mosquito. The standard diet was on hatching day: 0.04 mg Tetramin baby fish food per larva; 1 day after hatching: 0.06 mg; 2 days: 0.08 mg; 3 days: 0.16 mg; 4 days: 0.32 mg; 5 days or later: 0.6 mg. The low diet was 50% of the standard. Larvae were reared at constant temperatures, at 21, 25 or 29 °C, 70 ± 5% RH and a 12:12 h light:dark cycle

Longevity ranged from 26.4 days (± 2.54) for mosquitoes reared at 21 °C and standard food to 30.6 days (± 1.56) for mosquitoes reared at 29 °C and low food (Fig. 3). However, longevity was not significantly influenced by temperature, larval nutrition, or their interaction (Cox proportional hazards: all *P* > 0.35).

**Fig. 4:**
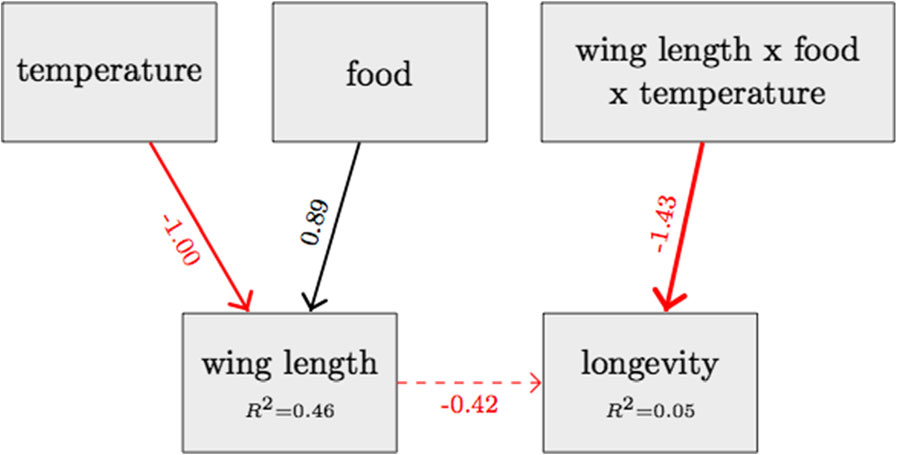
Structural equation model exploring the relationships between larval rearing temperature and nutrition level, wing length of emerged females, and their longevity. Positive relationships between variables are indicated with black arrows; negative relationships with red arrows. For clarity, only the significant paths are indicated (except for the effect of wing length on longevity, dotted arrow). The thickness of the paths is scaled according to the regression coefficient associated with each path. The numbers next to each arrow are standardised regression coefficients of the SEM (Table 2). Note that for the three-way interaction, the number shows the difference of the slope of longevity on wing length between, on the one hand, standard food and the highest temperature and, on the other hand, low food and the lowest temperature. The *R*^*2*^ associated with each component model is given with the relevant response variable

The piecewise structural equation model, fitting our data satisfactorily (Fisher’s *C* = 2.3, *df* = 2, *P* = 0.32), confirmed the direct effects of food and temperature during larval development on body size, and confirmed the lack of direct effects of food and temperature on longevity (Table 2 and Fig. 4).

**Table 2:** Standardized regression coefficients of the structural equation model

Response	Predictor	Estimate	SE	*P*-value
Wing length (wl)	Food (standard)	0.89	0.11	**<0.001**
Wing length (wl)	Temperature (29)	-1.00	0.14	**<0.001**
Wing length (wl)	Temperature (25)	-0.12	0.14	0.41
Longevity	wl * food (standard) * temperature (29)	-1.43	0.66	**0.031**
Longevity	wl * food (standard)	0.84	0.54	0.12
Longevity	Food (standard)	-0.56	0.41	0.16
Longevity	wl * food (standard) * temperature (25)	-0.88	0.64	0.16
Longevity	wl * temperature (29)	0.68	0.56	0.23
Longevity	Food (standard) * temperature (25)	0.54	0.50	0.28
Longevity	Temperature (29)	0.42	0.45	0.36
Longevity	wl * temperature (25)	0.50	0.57	0.38
Longevity	wl	-0.42	0.50	0.40
Longevity	Temperature (25)	0.03	0.38	0.94
Longevity	Food (standard) * temperature (29)	0.03	0.54	0.96

The estimate column gives the standardized regression coefficient associated with the predictors, or their interactions (For example, it shows the difference of the regression coefficients of longevity on size for certain foods and temperature, for the triple interactions). The *P*-values in bold text are inferior to 0.05 and indicate significant predictors

**Fig. 5:**
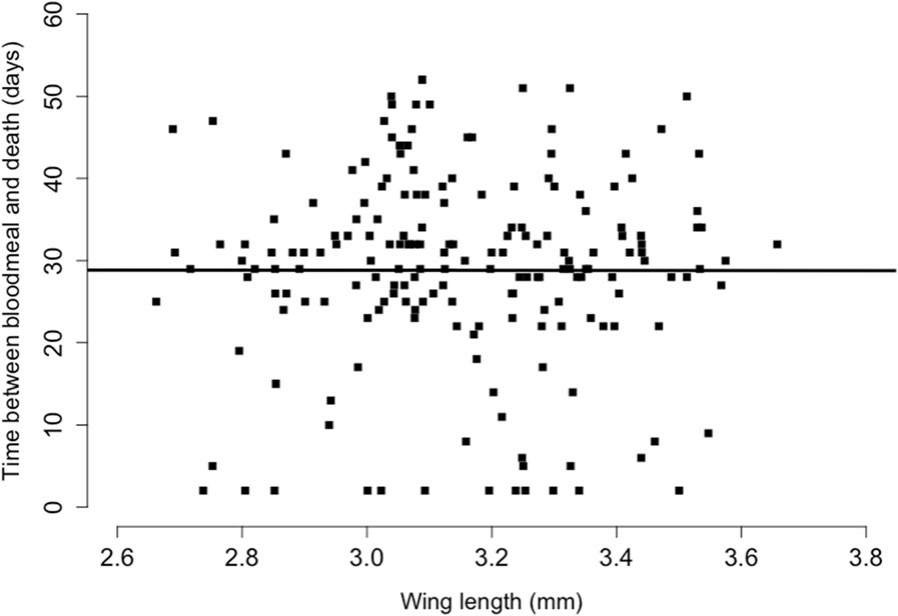
Mosquito longevity (in days) as a function of mosquito wing length (in mm). Each point shows the time between the blood meal and death of a female mosquito (longevity). The solid line represents the regression of longevity on wing length

**Fig. 6:**
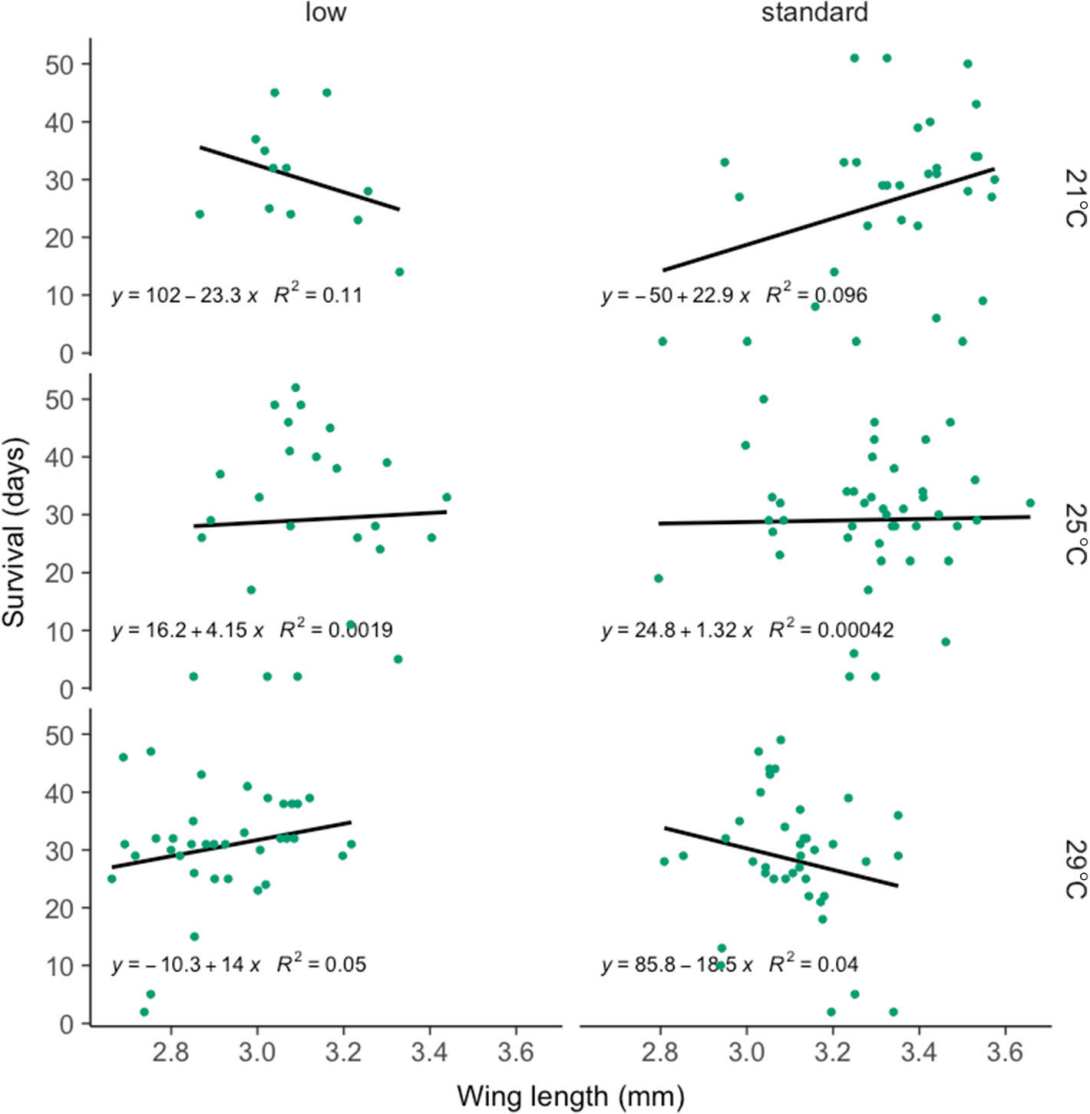
Mosquito longevity (in days) as a function of wing length (in mm) by larval rearing temperature (21, 25 and 29 °C) and level of larval nutrition (low, standard). Longevity is here the number of days between the blood meal and death for each female mosquito. The standard diet was on hatching day: 0.04 mg Tetramin baby fish food per larva; 1 day after hatching: 0.06 mg; 2 days: 0.08 mg; 3 days: 0.16 mg; 4 days: 0.32 mg; 5 days or later: 0.6 mg. The low diet was 50% of the standard. Larvae were reared at constant temperatures, at 21, 25 or 29 °C, 70 ± 5% RH and a 12:12 h light:dark cycle. The equation of the regression of longevity on wing length is given in each panel along with the *R*^2^ value

There were, however, indirect effects, which differed among environments. Thus, although overall no significant relationship was apparent between longevity and wing length (Figs. 4 and 5), there was a significant three-way interaction between wing length, food and temperature (Table 2). At 25 °C, longevity was only slightly related to wing length. At 29 °C longevity was positively related to wing length at low food but negatively at standard food, whereas at 21 °C the slope was negative at low food but positive at standard food (Fig. 6). No other pathways were significant (see Table 2).

## Discussion

While the overall relationship between mosquito size and longevity was weak, this relationship was apparent within individual larval treatments with the correlation between the two traits ranging from positive to negative among different treatments. At 25 °C, the relationship was weak. At 29 °C the relationship was negative at standard food and positive with low food. At 21 °C it was negative at low food and positive at standard food. Thus, this confirms our hypothesis that the relationship between size and longevity is not limited to a positive correlation and that the environment affects this relationship. The mechanisms underlying the changes of the slope and the direction of the correlation between environments are still unclear, and future experiments that delve into the physiological basis of this are warranted.

The increase in temperature decreases both the time to pupation and the adult size while a decrease in larval diet decreases mosquito size but increases development time. While we were not able to directly link adult size and development time in our data, it confirms that traits may react differently to the larval environment [4, 6–9].

One idea underlying the relationship between body size and longevity is that longevity is related to the teneral reserves carried over from the juvenile stages to adulthood. Consequently, larger mosquitoes should have more reserves [18] and therefore survive longer and it would be interesting to explore this further in the absence of adult nutrition or water availability. Variation in larval food affects adult mosquito reserves and resistance to desiccation with consequences for survival [30].

One explanation for the complexity of the relationship, instead of having just a positive correlation between size and longevity, is that temperature influences the teneral reserves and wing length differently during larval development. Indeed, in *Aedes albopictus* (Diptera: Culicidae) the relationship between teneral lipids and body size is linear with warm temperatures during larval development and exponential at lower temperatures [31]. In *An. gambiae* the relation between weight and wing length also varies with the temperature of the larval environment [32].

Another explanation is that the larval temperature and food levels influence the feeding behaviour of adults (blood and sugar consumption), which could have a large effect on longevity. There is, for example, a positive correlation between the larval food levels and the blood meal volume in *An. gambiae* [33]. The larval environment influences mosquito body size, and size affects the first meal choice of mosquitoes with smaller mosquitoes being more likely to take a sugar meal [34]. In addition, larger female mosquitoes accumulate reserves from blood meals more efficiently and need fewer blood meals to develop mature eggs [19]. Such differences in feeding regimes may also impact the effectiveness of vector control due to the variability in exposure to classical vector control tools like long-lasting insecticide-treated nets (LLIN) or indoor residual spraying (IRS) or new tools like attractive-toxic sugar baits (ATSB) [35], raising further the importance of understanding the influence of the larval environment.

Adult mosquitoes were kept at the constant temperature of 19 ± 1 °C, after the blood meal, which is at the lower part of the temperature range for adult *An. gambiae* mosquitoes and malaria transmission, 17 to 40 °C [36–39]. Repeating this study at higher or fluctuating temperatures may modify the relationship observed here, as constant and fluctuating adult temperatures are known to influence adult survival with increased longevity at lower temperatures [20, 40, 41]. The use of a low temperature with increased longevity is a conservative choice, as if the observed association between longevity and body size vary with different larval environments for a low adult temperature, they may just vary even more at higher temperatures.

Larvae were reared in the laboratory using larval diet based on previous studies [26, 42] and it is possible that even half our standard diet is still too comfortable for *An. gambiae* mosquitoes compared to natural conditions. Larvae are filter-feeders and what they feed on in natural conditions remains unclear [43]. The presence of algae increases mosquito density, especially for the last larval stages [44], and larvae prefer sunny sites with little aquatic vegetation that are favourable to algal growth [45]. Even if the laboratory diets have some differences with the hypothesised natural diets, we would then expect a greater effect of more variation in larval diets or harsher natural conditions on the correlations between adult body size and longevity.

Another important factor of vectorial capacity in addition to longevity or survival, is vector competence, which also depends on the body size of the vector and the larval environment [16]. For instance, smaller *Aedes aegypti* mosquitoes can be either more [46] or less [47] resistant to dengue virus. This relationship is influenced by larval density in both studies, but it changes from negative to positive by adding the effect of larval competition in the second study [47].

For evolutionary considerations, it would be important to also look at fecundity. Other studies suggest that the relationship between size and fecundity depends less on the environment: smaller mosquitoes are less fecund independently of temperature or food [9, 48]. However, how body size affects reproductive success (the combination of fecundity and longevity) in different environments is not known but would be critical for understanding the evolution of mosquito life history and the consequences on malaria transmission.

## Conclusions

To conclude, the relation between size and longevity differed among larval environments, with larger mosquitoes living longer in some environments but less long in others. This confirmed our hypothesis that the relationship between size and longevity is not limited to a positive correlation and that the interaction between environmental factors can change the slope and the direction of the relationship. A better understanding of this complexity and its mechanisms is necessary to understand and model the evolution of the life history traits of mosquitoes, the transmission of mosquito-borne diseases, and the influence on vector control.

## Additional files

Additional file 1: **Table S1.** Wing length and longevity per larval environment dataset. (XLS 42 kb)

Additional file 2: **Table S2.** Pupation time per larval environment dataset. (XLS 80 kb)

Additional file 3: Script for the piecewise SEM in R. The script is to be used with the Additional file 1. The first part of the script enables the user to install all the necessary packages and to import the dataset. The rest of the script enables the user to realise the piecewise SEM. The different steps are detailed from creating the model to obtaining the standardised regression coefficients. (R 1 kb)

## Abbreviations

GLM: Generalised linear model
IRS: Indoor residual spraying
LLIN: Long-lasting insecticide-treated nets
PBS: Phosphate-buffered saline
SE: Standard error
SEM: Structural equation model
